# The Role of Pt_3_/TiO_2_(*h K l*) Interface in Hydrogen and Water Splitting: A DFT Perspective on Reactivity and Limitations

**DOI:** 10.1021/acsomega.5c01650

**Published:** 2025-08-07

**Authors:** Ericson H. N. S. Thaines, Aline C. Oliveira, Leandro A. Pocrifka, Gustavo Doubek, Leonardo M. da Silva, Hudson Zanin, Renato G. Freitas

**Affiliations:** † Laboratory of Electrochemistry and Energy, Department of Chemistry, 67826 Federal University of Amazonas, Manaus, Amazonas 69067-005, Brazil; ‡ Laboratory of Computational Materials, Institute of Physics & Institute of Chemistry, Federal University of Mato Grosso, Cuiaba, Mato Grosso 78060-900, Brazil; § Laboratory of Advanced Batteries, Advanced Energy Storage Division,Center for Innovation on New Energies, School of Chemical Engineering, 529941 University of Campinas, Campinas, São Paulo 13083-852, Brazil; ∥ Department of Chemistry, Laboratory of Fundamental and Applied Electrochemistry, Federal University of Jequitinhonha e Mucuri’s Valley, Rodovia MGT 367, km 583, 5000, Alto da Jacuba, Diamantina, Minas Gerais 39100-000, Brazil; ⊥ Advanced Materials Laboratories and Manufacturing Group, Advanced Energy Storage Division, Center for Innovation on New Energies, University of Campinas, Campinas, São Paulo 13083-852, Brazil

## Abstract

The Pt/TiO_2_ interface has shown promise as a photocatalyst for hydrogen evolution reactions (HER). However, understanding hydrogen and water splitting reactions on the Pt surface of the Pt/TiO_2_ interface remains a significant challenge. The Pt_3_/TiO_2_(*h k l*) interface was characterized using X-ray diffraction (XRD) with Rietveld refinement analysis, which revealed reflections attributed to Pt­(*1 1 1*) and anatase TiO_2_(*h k l*). Theoretical modeling of the Pt_3_/TiO_2_(*h k l*) interface consists of approximately 60% TiO_2_ and 40% Pt, as determined by Rietveld refinement. The electronic properties were obtained using density functional theory (DFT)/plane-wave calculations on a model consisting of 39 atoms. The band structure and projected density of states (PDOS) of Pt_3_/TiO_2_(*h k l*) showed a new state between the valence and conduction bands, with contributions from the Pt 5*d* state, indicating metallic behavior. The initial steps of hydrogen and water splitting, as well as the transition states, were determined using the nudged elastic band (NEB) method for the reactions $H_{2}\to H_{ads}^{*}+H_{ads}^{*}$ and $H_{2}O_{ads}\to OH_{ads}^{*}+H_{ads}^{*}$ on the Pt_3_ surface of the Pt_3_/TiO_2_(*h k l*) interface. The Pt_3_/TiO_2_(*1 0 1*) interface exhibited the lowest activation energy (0.19 eV) for hydrogen molecule splitting, an exothermic reaction. Both Pt_3_/TiO_2_(*h k l*) interfaces exhibited an activation energy of approximately 1.4 eV for water splitting, an endothermic reaction. Therefore, hydrogen molecule splitting on the Pt_3_ surface is favorable, whereas water splitting is not, which may limit the hydrogen production rate (HGR) compared to other catalysts.

## Graphical abstract

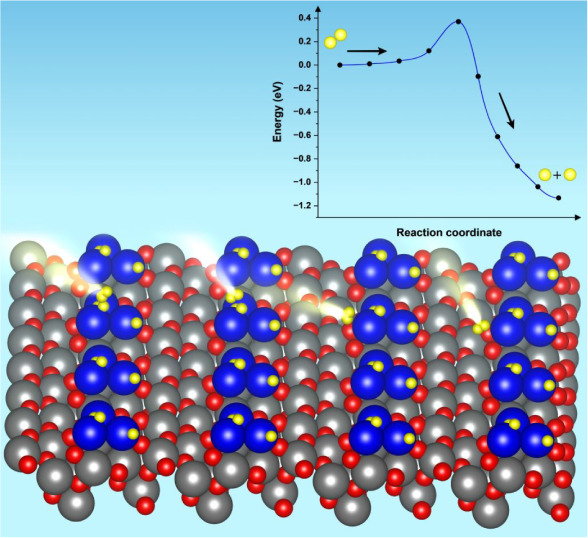

## 1 Introduction

The use of fossil fuels has significantly contributed to global climate change.^1,2^ As a result, many studies have concentrated on developing renewable energy sources as alternatives.^3,4^ Hydrogen fuel is a promising renewable energy source, especially for vehicle applications.^5^ Currently, the majority of hydrogen production relies on fossil fuels,^6^ but it can be obtained by photocatalysis^7^ or electrocatalysis.^8^ Hydrogen evolution reaction (HER) can be achieved via water splitting through photocatalysis,^9^ photoelectrochemical,^10^ thermal decomposition,^11^ and other methods. Many materials have been investigated and engineered as HER catalysts. Platinum (Pt)–based catalysts are widely known as among the most efficient for HER.^12–14^ McCrum and Koper^15^ studied HER kinetics on platinum through experimental and theoretical approaches. The authors^15^ proposed the rate-determining step of water splitting on platinum for HER:

$$H_{2}O+{\;}^{*}+e^{-}\to H^{*}+OH^{*}$$

where H* and OH* are adsorbed species. Therefore, hydrogen and hydroxyl adsorption on platinum are essential steps for HER. However, the use of platinum as a catalyst is restricted by its high cost and limited availability.^16^ Thus, alternative catalysts, including Pt/oxide-based materials, have been investigated as potential substitutes for Pt catalysts.^17–19^

Due to its catalytic and photocatalytic properties, the Pt/TiO_2_ interface is studied as an alternative catalyst.^20,21^ The metal/oxide interaction forms a Schottky junction,^22^ such as the interaction between Pt and an *n*-type semiconductor (TiO_2_). Platinum plays an important role in tuning the catalytic properties of TiO_2_ while reducing Pt usage, making it a cost-effective catalyst.^23,24^ In reactions driven by photocatalysis, charge carriers—electrons (*e* ^–^) and holes (h^+^)—are essential, but the quick recombination of *e* ^–^/*h* ^+^ carriers can limit the reaction. In this sense, the Pt/TiO_2_ interface can suppress or prolong the *e* ^–^/*h* ^+^ recombination, mainly through changes in the electronic structure of TiO_2_.^25,26^ In addition, the Pt/TiO_2_ interface’s photocatalytic activity is also influenced by other factors, such as the crystalline phase and surface on which the reaction occurs.

Nørskov et al.^27^ studied cyclic voltammograms for hydrogen on Pt­(*1 1 1*) and Pt­(*1 0 0*) from DFT/PW/PBE-GGA and observed that the differential binding energy for hydrogen in 0.25 ML coverage is −0.45 eV and −0.60 eV for Pt­(*1 1 1*) and Pt­(*1 0 0*) surfaces, respectively. These results indicate hydrogen’s interaction with Pt depends on the adsorption plane. Marković et al.^28^ performed experimental studies on the kinetics of the HER. The authors^28^ determined that the HER follows the Tafel reaction on the Pt­(*1 1 0*) surface and the Heyrovsky reaction on the Pt­(*1 0 0*) surface but did not determine the main mechanism on the Pt­(*1 1 1*) surface. In this sense, Nørskov et al. obtained DFT calculations for the HER in an electrochemical double layer on the Pt(111) electrode. The energy barrier for the Tafel reaction (2H*_ads_* → H_2_) was found to be 0.55 eV, whereas for the Heyrovsky reaction (H*_ads_* + H^+^ + *e* ^–^ → H_2_)), it was 0.35 eV, suggesting that the Heyrovsky reaction is probably the dominant mechanism. Since the activation energies of the Tafel and Heyrovsky reactions are relatively close, both mechanisms may operate in parallel.

Several studies have investigated the importance of the Pt­(*1 1 1*) surface by the adsorption of hydrogen on Pt­(*1 1 1*).^29–31^ Sugino et al.^32^ reported the accuracy needed to describe H adsorption, using DFT at the dRPA, PBE, and vdW-DFT levels of theory to determine the adsorption energy (*E*_*ads*_) of H on Pt­(*1 1 1*). The *E*_*ads*_ values obtained using dRPA and PBE were in good agreement, particularly when the experimental lattice constant was used. This suggests that PBE can reliably predict the relative stability of hydrogen adsorption at the face-centered cubic (H_*fcc*_) and hydrogen adsorption at the top (H_*top*_) sites on the Pt­(*1 1 1*) surface.

The Pt/TiO_2_ interface is extensively studied for its application in the HER. However, previous studies have primarily examined the adsorption of hydrogen on Pt rather than the splitting of molecular hydrogen on Pt, which is the main focus of this study. We analyzed the behavior of Pt_3_/TiO_2_(*h k l*) interfaces and investigated the influence of the Pt_3_ cluster phase and TiO_2_ surface on their structural and electronic properties. Structural analysis was performed using X-ray diffraction with Rietveld refinement, while electronic properties were investigated via density functional theory/plane wave (DFT/PW) calculations. This study provides novel insights into hydrogen and water splitting on the Pt_3_/TiO_2_(*h k l*) interface.

## 2 Theoretical Details

The theoretical modeling of the Pt_3_/TiO_2_(*h k l*) interface was based on experimental data obtained from a polymeric resin synthesized via the Pechini method and characterized by X-ray diffraction with Rietveld refinement^33^ (see Figure S1). The crystallographic parameters estimated through Rietveld refinement were used to calculate the electronic and structural properties and simulate reactions on the Pt_3_/TiO_2_(*h k l*) interface. The theoretical calculations were conducted using density functional theory^34,35^ plane-wave (DFT/plane-wave) implemented on the Quantum ESPRESSO^36^ Package. The electronic and structural properties were calculated using the electronic exchange and correlation interactions described by the functional developed by Perdew–Burke–Ernzerhof^37^ (PBE) and ultrasoft pseudopotentials proposed by Vanderbilt, with electrons from O (2*s*, 2*p*), Ti (3*s*, 3*p*, 4*s*, 3*d*), and Pt (4*f*, 5*d*, 6*s*) shells. A convergence test was performed to optimize the system, setting the plane-wave basis set energy cutoff at 55 Ry for the smooth wave functions and 550 Ry for the charge density. The electronic structure calculations employed a DFT/PBE+U approach with the Hubbard correction. The effective *U* parameter was set at *U*_*eff*_ = 3.5 eV for *d* – Ti and *p* – O, considering DFT/PBE+*U*_*d*_+*U*_*p*_. For the Brillouin zone, integrations were performed 4 × 4 x 2 *k*-points (Pt_3_/TiO_2_(*0 0 1*) interface), and 2 × 4 × 1 *k*-points (Pt_3_/TiO_2_(*1 0 1*) interface) mesh sampling based on the Monkhorst–Pack scheme.^38^ The Pt_3_/TiO_2_(*h k l*) interface was modeled using a 2 × 2 slab for the (*1 0 1*) and (*0 0 1*) planes, with 36 TiO_2_ (anatase) atoms and 3 Pt atoms in the (*1 1 1*) plane (see Figure S2). The phase composition was approximately 60% TiO_2_ and 40% Pt, as determined by Rietveld refinement.

The Pt_3_/TiO_2_(*h k l*) interfaces were based on the orthorhombic system, and the electronic band structures (BS) were calculated using the *k*-points path reported by Curtarolo et al.^39^ The hydrogen and water splitting was simulated with the climbing nudged elastic band method (CI-NEB), with the k-points grid set at the Γ point. The *d*-band structure analysis was calculated from the Projected Density of States (PDOS) for Ti and Pt *d-*band center, where the *d-*band center (*ε*_*d*_) can be calculated from

$$\varepsilon_{d}=\frac{\int_{-\infty }^{E_{F}}E_{n_{d}}\left(E\right)dE}{\int_{-\infty }^{E_{F}}n_{d}\left(E\right)dE}$$

where *n*_*d*_(E) is the density of states of Ti and Pt *d*-orbitals obtained from PDOS. The atomic coordinates (*x y z*) determined by Rietveld refinement and used in the theoretical calculations are shown in Supporting Information.

## 3 Results and Discussion

### 3.1 Computational Modeling for Pt_3_/TiO_2_(*h k l*) Interface

The Pt_3_/TiO_2_(*h k l*) interface modeling was based on the X-ray diffraction (XRD) and Rietveld refinement, as shown in Figure S1 and Table S1. The XRD analysis revealed reflections corresponding to the platinum (Pt) and titanium dioxide (TiO_2_) phases, with reflections at 39.7178 (*1 1 1*)-*hkl* surface associated with the Pt crystalline phase (Pt-CIF ICSD 243678) and reflections at 25.2978 (*1 0 1*)-*hkl* surface and 37.6865 (*0 0 4*)-*hkl* surface to the anatase TiO_2_ crystalline phase (TiO_2_-CIF ICSD 93098). The weight fraction analysis indicated that TiO_2_ constituted ∼60% of the material, whereas Pt contributed ∼40%. Thus, Density Functional Theory (DFT) calculations were performed to model Pt_3_/TiO_2_(*0 0 1*) and Pt_3_/TiO_2_(*1 0 1*), using weight fractions determined by Rietveld refinement, Figure 1a,b.

**Figure 1:**
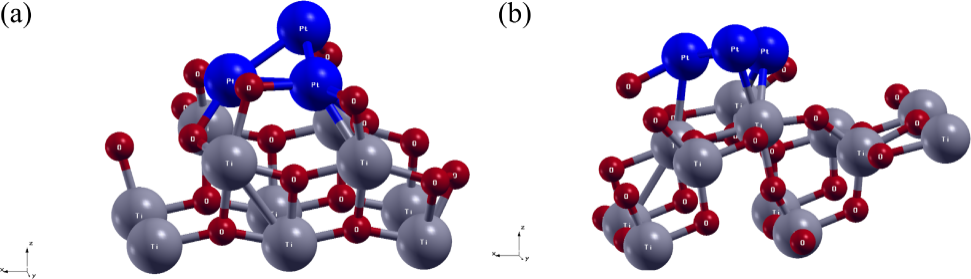
Optimized structures of (a) Pt_3_/TiO_2_(0 0 1) interface and (b) Pt_3_/TiO_2_(1 0 1) interface. The red balls represent the oxygen atoms, the gray balls represent the titanium atoms, and the blue balls represent the platinum atoms.

The Pt_n_ clusters interacted strongly with oxide support such as TiO_2_, produced a strong metal–support interaction. The Pt_3_ cluster exhibits stable triangular configurations when adsorbed on the anatase TiO_2_,^40,41^ indicating a Pt_3_/TiO_2_ stable interface for catalytic properties. Additionally, the Pt_3_ cluster can present many distinct H binding sites with varying stabilities, resulting in different reaction channels. These reaction channels may involve H atom diffusion, H–H molecule restructuring on the surface, and H_2_ molecule desorption.^42^ Therefore, the Pt_3_ cluster is a suitable theoretical model for the Pt/TiO_2_ interface, enabling detailed investigations of hydrogen and water splitting on the Pt_3_/TiO_2_ (*h k l*) surface.

The bond lengths and types significantly influence the structural and electronic properties of the Pt_3_/TiO_2_(*h k l*) interface. As shown in Figure 1a,b, the Pt–Pt, Pt–Ti, and Pt–O bonds at the interface can be observed, and the analysis of bond lengths between atoms is pivotal for understanding interface behavior. All the bond lengths of the optimized structures of the Pt_3_/TiO_2_(*h k l*) interface can be observed in Figure S3 and Table S2. For the Pt_3_/TiO_2_(*0 0 1*) interface, Figure 1a, and Pt_3_/TiO_2_(*1 0 1*) interface, Figure 1b, the Pt–Pt bond lengths in the Pt_3_ cluster range from 2.4817 Å up to 2.6397 Å. The Pt–Pt bond lengths in the Pt_3_ cluster range from 2.4817 Å to 2.6397 Å, which aligns with reported values for Pt clusters and oxide-supported Pt, typically ranging from 2.50 Å to 2.80 Å.^43–48^ The Pt_3_/TiO_2_(*0 0 1*) interface exhibits a single Pt–Ti bond of the 2.6203 Å, involving a five-coordinated Ti (Ti_5C_) atom. In contrast, the Pt_3_/TiO_2_(*1 0 1*) interface features four Pt–Ti bonds, ranging from 2.6372 Å to 2.8049 Å, involving both four-coordinated Ti (Ti_4C_) and five-coordinated Ti (Ti_5C_) atoms. The Pt_3_/TiO_2_(*0 0 1*) interface presented six Pt–O bonds ranging from 1.8569 Å up to 2.0568 Å, and the Pt_3_/TiO_2_(*1 0 1*) interface two Pt–O bonds with 2.0206 Å and 2.0643 Å. Overall, the observed bond lengths in the Pt_3_/TiO_2_(*h k l*) interfaces agree with previously reported values in the literature.^48–51^

### 3.2 Electronic Structure

The electronic structure of the Pt_3_/TiO_2_(*h k l*) interface was investigated by analyzing the band structure (BS) and projected density of states (PDOS), as shown in Figure 2a,b.

**Figure 2:**
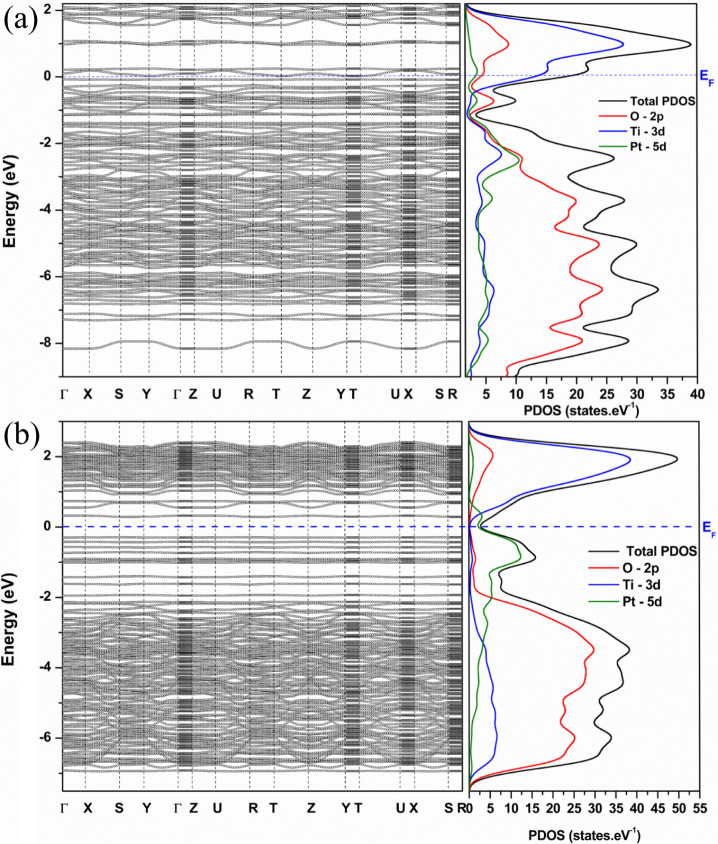
Band structures (BS) and projected density of states (PDOS) for (a) Pt_3_/TiO_2_(*0 0 1*) interface and (b) Pt_3_/TiO_2_(*1 0 1*) interface.

The BS and PDOS calculated for the Pt_3_/TiO_2_(*h k l*) interface indicate metallic behavior in both interfaces, where overlapping valence and conduction bands are observed. Anatase TiO_2_ exhibits semiconductor behavior for the isolated phase, with an indirect band gap (M → Γ/*k* – path) calculated at 3.00 eV, while the experimentally determined band gap is 3.20 eV.^52^ Additionally, the platinum phase exhibits metallic behavior, as shown in Figure S4. The PDOS behavior of the Pt_3_/TiO_2_(*h k l*) interfaces is similar to that of the anatase TiO_2_ phase, as shown in Figure 2a,b. In both interfaces, the O–2*p* states are localized in the valence band (VB) below the Fermi energy (E_F_), while the Ti–3*d* states are in the conduction band (CB) above E_F_, compared to the PDOS of anatase TiO_2_ (Figure S4a). The metallic behavior observed in the Pt_3_/TiO_2_(*h k l*) interfaces is attributed to a new state between VB and CB. In the Pt_3_/TiO_2_(*0 0 1*) interface (Figure 2a), the O–2*p* and Pt–5*d* states are localized in this intermediate state between VB and CB, attributed to the Pt–O bonds present at the interface. However, in the Pt_3_/TiO_2_(*1 0 1*) interface (Figure 2b), the Pt–5*d* states are localized in this intermediate state, as this interface exhibits Ti–Pt bonds in its interface. Jin et al.^53^ studied single metal/TiO_2_ interfaces and their photocatalytic properties using DFT at the PBE exchange-correlation level. According to the authors,^53^ the electronic band structure of the Pt/TiO_2_(1 0 1) interface presented new bands between VB and CB, associated with a single platinum metal. Wang et al.^54^ investigated the gas-sensing properties of the Pt_3_/TiO_2_(*1 0 1*) interface, calculating its electronic structure using DFT at the PBE level. Their results revealed states between VB and CB, showing a similar behavior to that observed by Jin et al.^53^

The emergence of states between VB and CB in the Pt_3_/TiO_2_(*h k l*) interfaces highlights the different bonding configurations on the electronic structures, as observed in the BS and PDOS. These bonding within the optimized Pt_3_/TiO_2_(*h k l*) interfaces are further reflected in the charge density distribution (Δ*n*) and electron localization function (ELF – η­(*r*)), as shown in Figure 3.

**Figure 3:**
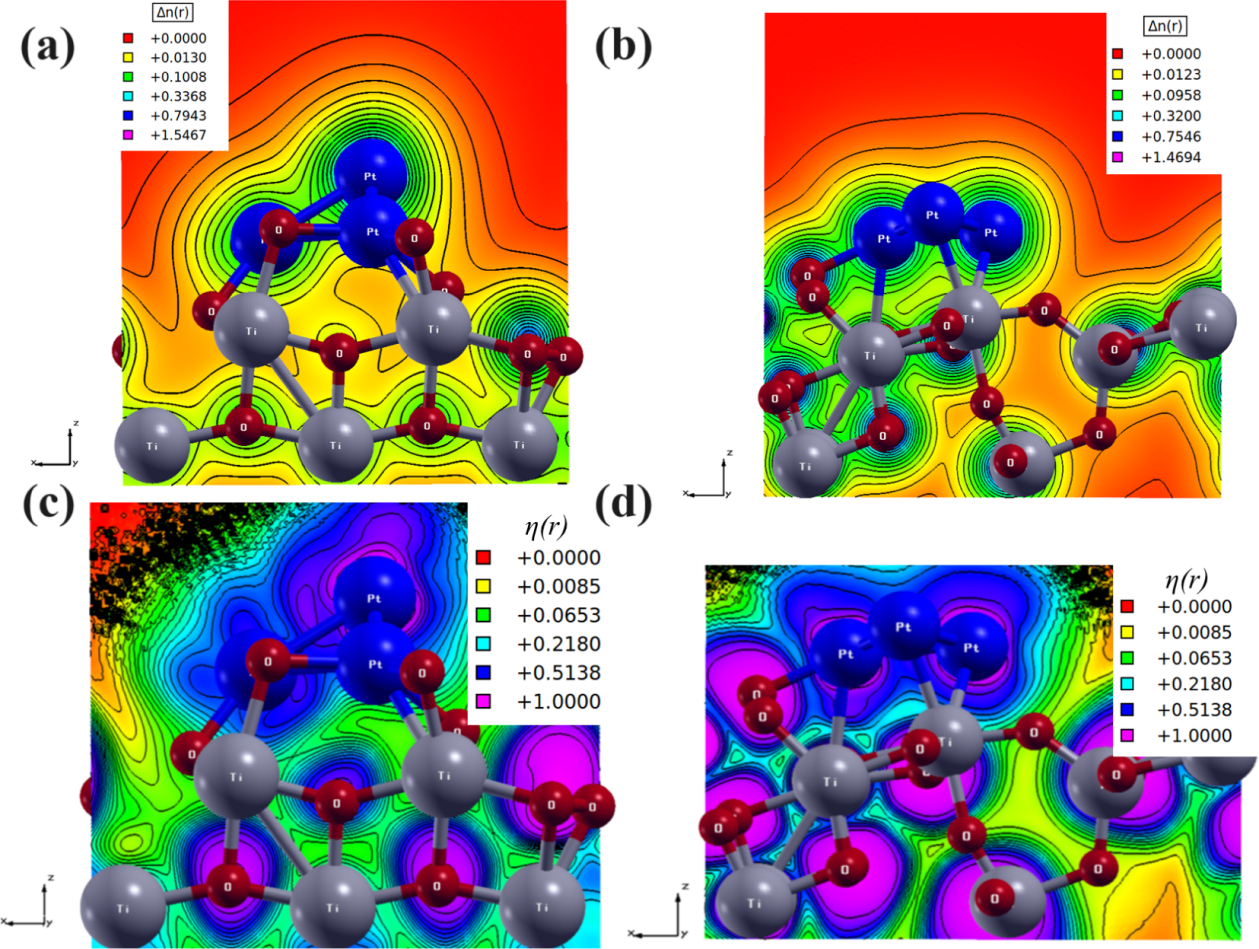
2D charge density distribution (Δn) and 2D electron localization function (ELF – η­(r)) of the (a)–(c) Pt_3_/TiO_2_(0 0 1) interface and (b)–(d) Pt_3_/TiO_2_(1 0 1) interface.

In Figure 3a–d, the 2D charge density distribution (Δ*n*) and electron localization function (ELF – η­(*r*)) are mainly localized on oxygen atoms in the TiO_2_ phase and the Pt atoms in the Pt phase for both interfaces. The Pt_3_/TiO_2_(*0 0 1*) interface (Figure 3a) exhibits maximum charge density (Δ*n*_max_) values of approximately 1.54, while the Pt_3_/TiO_2_(*1 0 1*) interface (Figure 3b) presents values around 1.47. The variation in Δ*n*_max_ indicates that the electronic structure of the Pt_3_/TiO_2_(*h k l*) interfaces is influenced by the TiO_2_(*0 0 1*) and TiO_2_(*1 0 1*) surfaces. Analyzing the 2D ELF – η­(*r*) maps, the Pt_3_/TiO_2_(*0 0 1*) interface (Figure 3c) displays Ti–O bonds with ionic characteristics, exhibiting η­(*r*) values between ∼0.06 and ∼0.2. The Pt–O bonds show a more covalent character compared to the Pt–Ti bond, with the η­(*r*) values ∼0.5 and ∼0.2, respectively. In contrast, the Pt_3_/TiO_2_(*1 0 1*) interface (Figure 3d) demonstrates a more covalent character in the Ti – O bonds compared to the Pt_3_/TiO_2_(*0 0 1*) interface, with η­(*r*) values ranging from ∼0.2 to ∼0.5. Similarly, the Pt–O bonds exhibit a stronger covalent character than the Pt – Ti bonds, with the η­(*r*) values ∼0.5 and ∼0.2, respectively. The overlapping isoline basins observed in both interfaces further confirm the bonds observed.

The anatase TiO_2_ phase has been extensively discussed in the literature,^55–59^ demonstrating that the Δ*n* localizes on the oxygen atoms due to the differing electronegativity values of oxygen and titanium atoms (O = 3.5, Ti = 1.32 on the Pauling scale.^60^ However, the description of Δ*n* for the Pt_3_/TiO_2_(*h k l*) interface depends on several factors, including the type of interface, the use of clusters to represent the Pt phase, the crystalline structure of the Pt phase, whether as a single Pt atom or cluster, along with variations in the TiO_2_(*h k l*) planes. The Pt/TiO_2_ interface can be described using a single Pt atom on the TiO_2_(*1 0 1*), as reported by Zhang et al.^61^ The DFT analysis of Δ*n* at the interface indicated charge accumulation on oxygen atoms and the overlapping Δ*n*-basins in the Ti–O bonds, suggesting a covalent character. Additionally, the single Pt atom exhibited a gradual accumulation of charge, a behavior that can be associated with the adsorption of sulfur dioxide molecules on the TiO_2_ surface. In contrast, Wu et al.^62^ investigated the Pt/TiO_2_ interface using Pt clusters, with application on electrocatalytic hydrogen evolution. The authors^62^ constructed Pt_4_/TiO_2_(*1 0 1*) and Pt_4_/TiO_2_(*1 0 1*)­ $V_{o}^{*}$ interfaces using DFT calculations to analyze the electronic structure. The Pt_4_ cluster formed a pyramid-like structure, with Δ*n* localized on oxygen and Pt atoms near the TiO_2_ surface in the Pt_4_/TiO_2_(*1 0 1*)­ $V_{o}^{*}$ interface. The Pt atom farthest from the TiO_2_ surface displayed Δ*n* ∼ 0, while the interface Δ*n* distribution (0 < Δ*n* < 0.7) indicated a covalent character. Another approach to modeling the Pt/TiO_2_ interface was reported by Chen et al.,^49^ who constructed the Pt­(*0 0 1*) – fcc/TiO_2_(*0 0 1*) interface using a unit cell model based on the isolated phases. This resulted in two interface types: the first interface with Pt–O bonded interface, where Δ*n* localized on oxygen and Pt atoms near the TiO_2_ surface, and the second interface with Pt–Ti bonded interface, where increased Δ*n* localization was observed on Ti and Pt atoms. Therefore, several types of Pt/TiO_2_ interfaces can be studied or constructed, each influencing electronic properties and interfacial behavior, which may have implications for various applications.

### 3.3 Reaction Path of the Hydrogen Molecule Splitting on the Pt_3_/TiO_2_(*h k l*) Interfaces

Hydrogen molecule splitting was studied on the Pt_3_/TiO_2_(*h k l*) interfaces, considering their structural and electronic properties. The geometries of the interfaces, including the initial states (IS), transition state (TS), and final state (FS), were analyzed to understand the hydrogen splitting mechanism on the Pt_3_/TiO_2_(*0 0 1*) and Pt_3_/TiO_2_(*1 0 1*) interfaces, as shown in Figure 4.

**Figure 4:**
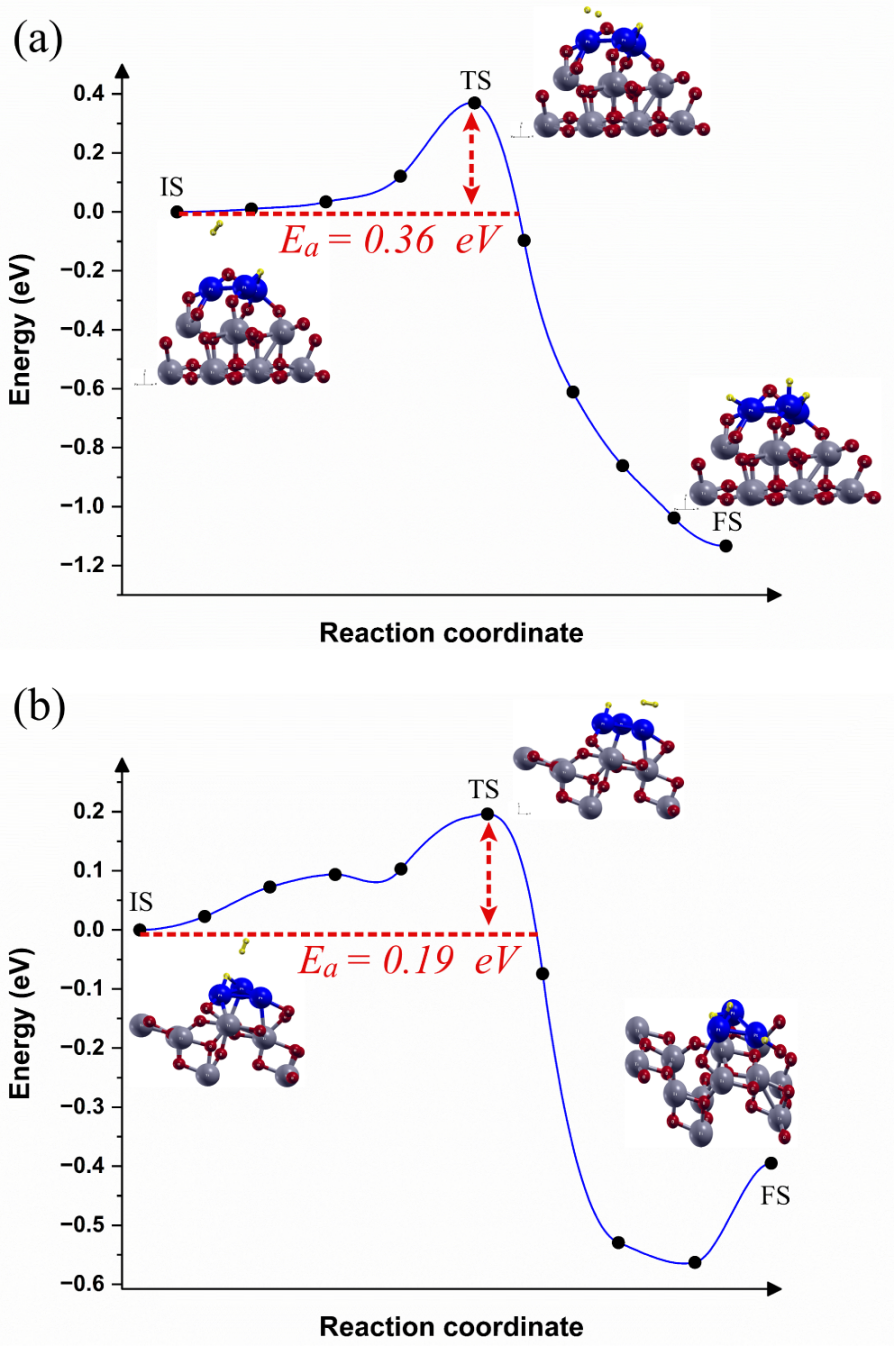
Reaction pathway and reaction barrier of single hydrogen molecule splitting (a) Pt_3_/TiO_2_(0 0 1) interface and (b) Pt_3_/TiO_2_(1 0 1) interface. The yellow balls represent the hydrogen atoms.

A single hydrogen molecule was selected to aim for a molecular scale understanding of the fundamental reaction pathway and to estimate the activation energy barrier for hydrogen molecule splitting, which was done using CI-NEB calculations with ten images-steps, where IS and FS were proposed based on a fundamental step from physisorbed H–H (H_2_) state to the dissociated state (H*):

$$H-H_{\mathrm{phy}}\to H_{ads}^{*}+H_{ads}^{*}$$

where $2H_{ads}^{*}$ are the species adsorbed on the Pt_3_/TiO_2_(*h k l*) interfaces. The activation energy barrier (*E_a_*)) for hydrogen molecule splitting can be determined using the following equation:

$$E_{a}=E_{TS}-E_{IS}$$

where *E_TS_* and *E_IS_* are the energies of transition and initial states, respectively. Additionally, the endothermic and exothermic nature of the reaction can be calculated by reaction energy (Δ*E*):

$$\Delta E=E_{FS}-E_{IS}$$

where *E_FS_* and *E_IS_* are the energies of the final and initial states, respectively.

The IS corresponds to the reactants, where the H_2_ molecule is physisorbed on the Pt_3_/TiO_2_(*h k l*) interface surfaces. The FS corresponds to the products, in which the hydrogen molecule has dissociated into $2H_{ads}^{*}$ species adsorbed on the interface surface. The H_2_ molecule dissociation on the Pt surface is known to be a spontaneous process.^63,64^ To simulate the initial spillover process,^65,66^ the H_2_ molecule was positioned toward the Pt surface, where one Pt atom already has an adsorbed hydrogen. In both Pt_3_/TiO_2_(*h k l*) interfaces, the H_2_ molecule orients toward Pt, with one of the hydrogen atoms positioned near the surface and the other farther away. This geometry is characteristic of an H_2_ molecule produced by the recombination of two desorbed hydrogen atoms from a metal/oxide surface.^67^ For the reaction path, different TS values were determined for the Pt_3_/TiO_2_(*0 0 1*) interface (Figure 4a) and the Pt_3_/TiO_2_(*1 0 1*) interface (Figure 4b). The H_2_ molecule dissociates on the Pt_3_/TiO_2_(*0 0 1*) interface, exhibiting an H- -H bond length of 0.8243 Å, and H- -Pt bond lengths between 2.0336 to 2.0889 Å. However, in the Pt_3_/TiO_2_(*1 0 1*) interface, the TS remains with the H_2_ molecule physisorbed on the surface, with a shorter H–H bond length of 0.7847 Å, and H- -Pt bond lengths between 2.1232 and 2.1537 Å.

The Δ*E* calculation indicates that the hydrogen molecule splitting is an exothermic process for both interfaces. The Pt_3_/TiO_2_(*0 0 1*) interface exhibits Δ*E* = −1.13*eV*, while the Pt_3_/TiO_2_(*0 0 1*) interface shows Δ*E* = −0.39*eV*. However, the *E*_a_ differs between the two interfaces, which *E*_a_ = 0.36 eV for Pt_3_/TiO_2_(*0 0 1*) interface and *E*_a_ = 0.19 eV for Pt_3_/TiO_2_(*1 0 1*) interface. Kwon et al.^68^ studied the Pt/TiO_2_ interface for H_2_ sensing using an electrical measurement system. The authors^68^ reported that the initial stage of H_2_ sensing involves the H_2_ molecules dissociation, with an activation energy of 36.4 kJ/mol (∼0.376 eV). The initial dissociation mechanism, converting H_2_ molecules into 2H* on the metal surface, was also reported by Wang et al.,^69^ who studied semihydrogenation under hydrogen spillover on the Pt/zeolite interface. Using the DFT/CI-NEB method was determined *E*_a_ = 0.36*eV* for the *H*_2_ → 2H* reaction on the Pt­(*1 1 1*)/zeolite interface. Although Pt_3_/TiO_2_(*h k l*) interfaces have been scarcely reported in the context of hydrogen molecule splitting, similar structures have been investigated. Wan et al.^70^ studied H_2_ adsorption, dissociation, and spillover on the Au_n_(*1 1 1*)/TiO_2_(*1 0 1*) interfaces. Through the DFT/CI-NEB method to simulate the H_2_ dissociation on the Au_3_(*1 1 1*)/TiO_2_(*1 0 1*) interface presented *E_a_* = 0.2*eV*. Therefore, the *E_a_* values determined in this study agree with the literature values, indicating that the Pt_3_/TiO_2_(*1 0 1*) interface is energetically more favorable for H_2_ molecule splitting than the Pt_3_/TiO_2_(*0 0 1*) interface. However, the Pt_3_/TiO_2_(*0 0 1*) interface demonstrates a more spontaneous reaction, as indicated by its exothermic reaction (smaller Δ*E*) compared to the Pt_3_/TiO_2_(*1 0 1*) interface.

The difference in *E_a_* and Δ*E* for the Pt_3_/TiO_2_(*h k l*) interfaces can be attributed to the electronic properties and the reaction mechanism, particularly the characteristics of the transition state (TS). As previously discussed, the Pt_3_/TiO_2_(*0 0 1*) interface presented a higher Δ*n*_max_ value compared to the Pt_3_/TiO_2_(*1 0 1*) interface, which can be associated with its more spontaneous reaction (lower Δ*E*). However, the smaller *E_a_* presented by the Pt_3_/TiO_2_(*1 0 1*) interface is likely influenced by differences in the TS – step and the PDOS structure, as shown in Figure 5.

**Figure 5:**
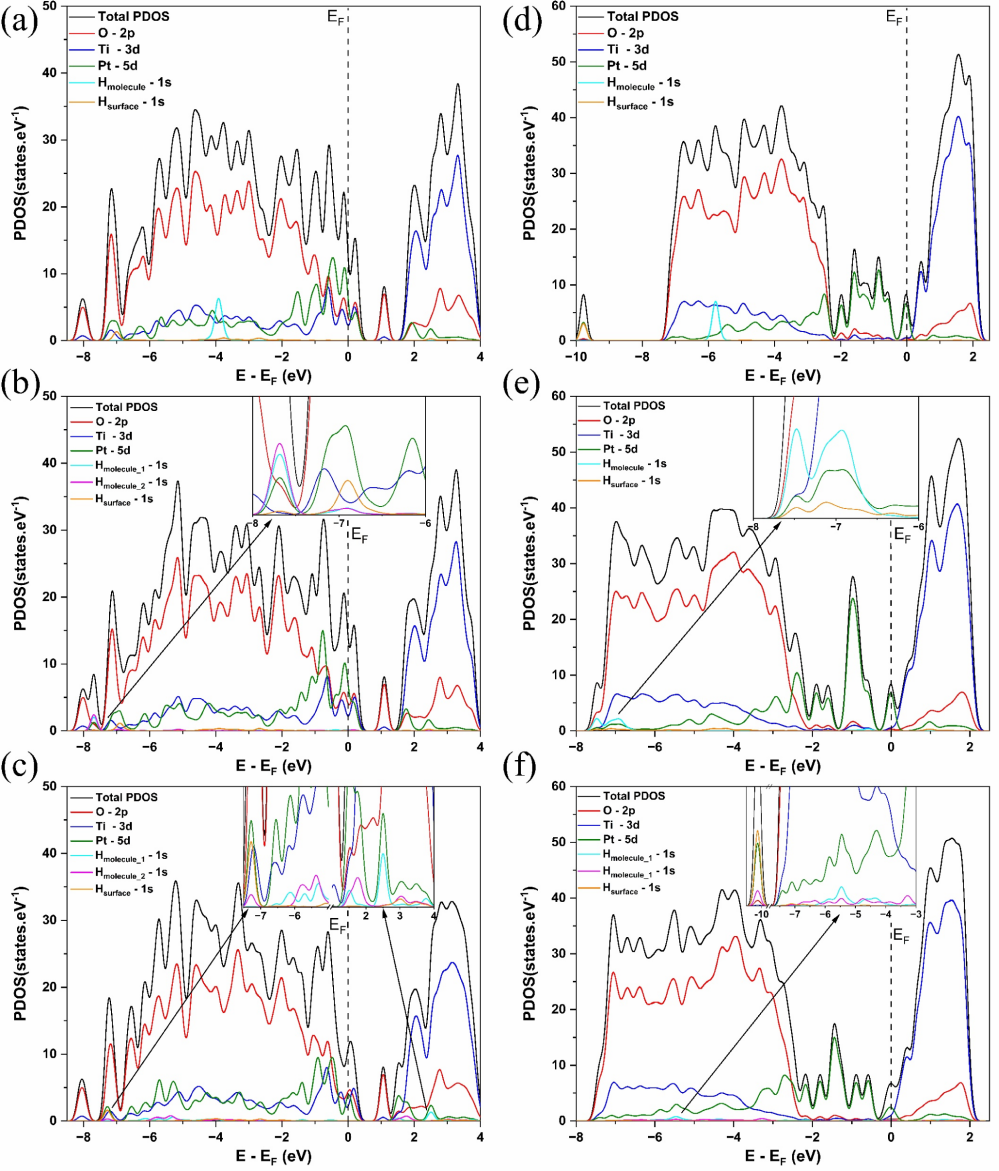
Projected density of states (PDOS) for hydrogen splitting on the Pt_3_/TiO_2_(0 0 1) interface (a) IS, (b) TS, (c) FS, and for Pt_3_/TiO_2_(1 0 1) interface (d) IS, (e) TS, and (f) FS.

It is possible to observe in Figure 5a–f that the Pt–5*d* states, green lines in PDOS, are localized near the Fermi energy (*E*_*F*_). In the initial state (IS) of the Pt_3_/TiO_2_(*0 0 1*) interface (Figure 5a), the H–1*s* state of the H_2_ molecule, cyan line in PDOS, is localized around −4.0 eV, while the H–1*s* state of the surface adsorbed H atom, orange line in PDOS, appears at approximately −7.0 eV. In contrast, for the IS of the Pt_3_/TiO_2_(*1 0 1*) interface (Figure 5d), the H–1*s* state of the H_2_ molecule is located near −6.0 eV, and the H–1*s* state of the surface adsorbed H atom appears at −10 eV. This energy difference suggests that hydrogen interaction in the IS is more stable in the Pt_3_/TiO_2_(*1 0 1*) interface.

In the transition state (TS) of the Pt_3_/TiO_2_(*0 0 1*) interface (Figure 5b), the H_2_ molecule is dissociated. The H–1*s* state of the H atoms from the H_2_ molecule is localized at a lower energy level, approximately −7.7 eV (inset graph, Figure 5b), with an overlapping Pt – 5d state. The H–1s state of the surface adsorbed H atom remains unchanged at −7.0 eV. On the other hand, in the TS of the Pt_3_/TiO_2_(*1 0 1*) interface (Figure 5e), the H_2_ molecule remains physiosorbed on the surface and is not dissociated. Its H–1*s* state is localized above −7.5 eV (inset graph, Figure 5e), with overlapping Pt–5d and H–1s states of the surface adsorbed H atom. Therefore, the TS of the Pt_3_/TiO_2_(*0 0 1*) interface exhibits lower energy states than the Pt_3_/TiO_2_(*1 0 1*) interface, indicating that more energy is required for the hydrogen molecule splitting in this interface. As a results, the Pt_3_/TiO_2_(*0 0 1*) interface presents a higher activation energy (*E_a_*)) for the reaction.

In the final state (FS) of the Pt_3_/TiO_2_(*0 0 1*) interface (Figure 5c), the H – 1s state of the H atoms from the H_2_ molecule is distributed across two energy regions: one lower energy level between −6.0 eV and −7.0 eV, and another higher energy level between 2.0 and 3.0 eV above *E*_*F*_. The H–1s state of the surface adsorbed H atom is localized slightly below −7.0 eV (inset graph, Figure 5c). For the FS of the Pt_3_/TiO_2_(*0 0 1*) interface (Figure 5f), the H–1s state of the H atoms from the H_2_ molecule appears at −6.0 eV and −5.0 eV. The H–1s state of the surface adsorbed H atom remains localized slightly below −10 eV, similar to its position in the IS (inset graph, Figure 5f). These lower energy states observed in the FS of the Pt_3_/TiO_2_(*0 0 1*) interface indicate a more spontaneous process, as the system reaches a more stable final state.

The PDOS structure of hydrogen molecule splitting on the Pt_3_/TiO_2_(*h k l*) interfaces provides insights into the relationship between *E_a_* and Δ*E* by revealing the energy level of electronic states. Additionally, analyzing the *d*-band behavior plays a fundamental role in understanding the reactivity of the Pt_3_/TiO_2_(*h k l*) interfaces, as it influences catalytic activity. Table 1 shows the *d*-band center (ε_*d*_), *d*-bandwidth (*w*_*d*_), and Fermi energy (*E*_*F*_) levels, for the isolated Pt_3_/TiO_2_(*h k l*) interfaces, as well as the initial state (IS), transition state (TS), and final state (FS) during the hydrogen molecule splitting process.

**Table 1:** Electronic Structure of the *D*-Band Projected: *D*-Band Center (ε_*d*_), *D*-Band Width (*w*_*d*_), and Fermi Energy (E_F_) of the TiO_2_, Pt Isolate Phase, and Hydrogen Splitting States on the Pt_3_/TiO_2_(*h K l*) Interface

	Pt_3_/TiO_2_(*0 0 1*)		Pt_3_/TiO_2_(*1 0 1*)	
*d*-band shape	Ti *d*-band	Pt *d*-band	Ti *d*-band	Pt *d*-band
*ε*_*d*_	–3.37	–2.93	–4.84	–2.05
*w*_*d*_	4.88	2.56	5.44	1.78
*E*_*F*_	–1.74		–0.44	
*Initial state*				
*ε*_*d*_	–4.40	–2.76	–6.62	–2.71
*w*_*d*_	6.25	2.92	6.94	2.36
*E*_*F*_	–1.96		–0.07	
*Transition state*				
*ε*_*d*_	–4.42	–2.96	–6.69	–2.32
*w*_*d*_	6.24	3.13	6.97	2.01
*E*_*F*_	–1.75		–0.01	
*Final state*				
*ε*_*d*_	–4.38	–3.32	–6.78	–2.97
*w*_*d*_	6.22	3.18	6.98	2.37
*E*_*F*_	–1.86		0.02	

Analyzing the isolated Pt_3_/TiO_2_(*h k l*) interfaces, the Pt_3_/TiO_2_(*0 0 1*) interface showed ε_*d*‑Ti_ and ε_*d*‑Pt_ energy levels of −3.37 eV and −2.76 eV, respectively. The Fermi energy (E_*F*_) was −1.96 eV, suggesting the presence of antibonding states above the E_*F*_, as the ε_*d*_ values are close to the Fermi level. In contrast, the Pt_3_/TiO_2_(*1 0 1*) interface exhibited ε_*d*‑Ti_ and ε_*d*‑Pt_ values of −4.84 eV and −2.05 eV, both well below the E_*F*_, indicating antibonding states below the E_*F*_. For the IS, a shift to lower energy levels was observed for both interfaces. The ε_*d*‑Pt_ of the Pt_3_/TiO_2_(*0 0 1*) interface shifted by 0.17 eV, while the Pt_3_/TiO_2_(*1 0 1*) interface showed a more significant shift by 0.66 eV. This larger shift suggests that the Pt_3_/TiO_2_(*1 0 1*) interface interacts more strongly with hydrogen molecules. In the TS, the Pt_3_/TiO_2_(*0 0 1*) interface exhibited a *w*_*d*‑Pt_ value of 3.13 eV, whereas the Pt_3_/TiO_2_(*1 0 1*) interface had a *w*_*d‑*Pt_ value of 2.01 eV. The ε_*d*‑Pt_ in the Pt_3_/TiO_2_(*1 0 1*) interface shifted to a higher energy level, indicating a smaller *E_a_* compared to the Pt_3_/TiO_2_(*0 0 1*) interface. The smaller *w*_*d*_ values reflect narrower *d*-band widths, suggesting more selective reactivity during the hydrogen splitting process, contributing to the reduction of *E_a_*. For the FS, the ε_*d*‑Pt_ for the Pt_3_/TiO_2_(*0 0 1*) interface was −3.32 eV, indicating a more stable state compared to the Pt_3_/TiO_2_(*1 0 1*) interface, which had an ε_*d*‑Pt_ value of −2.97 eV. Therefore, as shown in the PDOS structure (Figure 5c–f), the hydrogen molecule splitting on the Pt_3_/TiO_2_(*0 0 1*) interface corresponds to a more spontaneous process.

In summary, the hydrogen molecule splitting reaction path on the Pt_3_/TiO_2_(*h k l*) interfaces involved distinct reaction states. The initial state (IS) presented the physisorption of the H_2_ molecule on the Pt_3_/TiO_2_(*h k l*) interface, where the Pt surface influenced the spatial arrangement of the hydrogen molecule. Different transition states (TS) were determined for the Pt_3_/TiO_2_(*0 0 1*) and Pt_3_/TiO_2_(*1 0 1*) interfaces, indicating differences in bond lengths and adsorption geometries. The calculated Δ*E* values suggest that the hydrogen molecule splitting is exothermic for both interfaces. However, The *E_a_* is lower for the Pt_3_/TiO_2_(*1 0 1*) interface, indicating that this surface is more favorable for hydrogen dissociation. In contrast, the Pt_3_/TiO_2_(*0 0 1*) interface is more thermodynamically favorable, as suggested by the reaction energy (ΔE). The PDOS analysis provided insights into the interactions between hydrogen and Pt atoms across the IS, TS, and FS, highlighting the stability of the reaction state on the Pt_3_/TiO_2_(*0 0 1*) interface. The *d*-band analysis further revealed that the shift in ε_*d*_ increases surface reactivity, leading to a weaker H–H bond in the TS, which contributes to the lower *E_a_* observed for the Pt_3_/TiO_2_(*1 0 1*) interface.

### 3.4 Reaction Path of the Water Splitting on the Pt_3_/TiO_2_(h K L) Interface

Water splitting on the Pt_3_/TiO_2_(*h k l*) interfaces was studied by analyzing their structural and electronic properties. The reaction pathway was mapped through the initial states (IS), transition state (TS), and final state (FS), as shown in Figure 6. The geometries of these states reveal the adsorption configurations of the reaction mechanism. A single water molecule was chosen to provide molecular-scale insight into the fundamental steps of the reaction and to estimate the activation energy (*E*_a_) using CI-NEB calculations with nine image steps. The reaction follows the dissociation of adsorbed H_2_O into OH* + H*:

$$H_{2}O_{ads}\to OH_{ads}^{*}+H_{ads}^{*}$$

where OH^*^ and H^*^ are adsorbed species on the Pt_3_/TiO_2_(*h k l*) interfaces. The ΔE values suggest that water splitting exhibits different thermodynamic characteristics depending on the interface.

**Figure 6:**
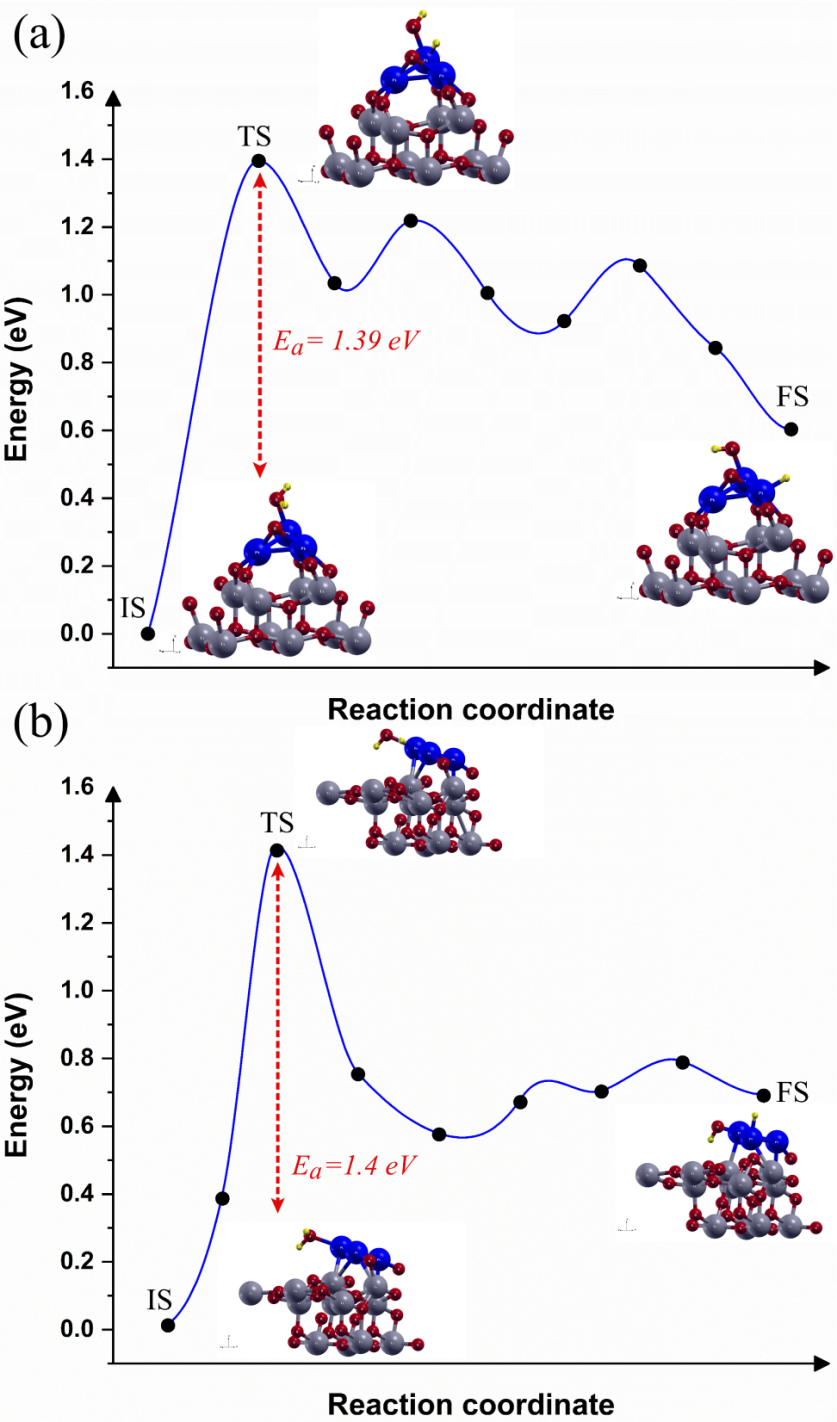
Reaction pathway and reaction barrier of water splitting (a) Pt_3_/TiO_2_(0 0 1) interface and (b) Pt_3_/TiO_2_(1 0 1) interface. The yellow balls represent hydrogen atoms.

The IS of splitting on the Pt_3_/TiO_2_(*h k l*) interfaces corresponds to the water molecule adsorbed on the interface surfaces. The FS corresponds to the dissociated water molecules, such as OH^*^, and H^*^, species adsorbed on the interface surface. In both Pt_3_/TiO_2_(*h k l*) interfaces, one of the H atoms from the water molecule is oriented toward the Pt surface, with one of the H atoms positioned near the TiO_2_(*1 0 1*) surface in the Pt_3_/TiO_2_(*1 0 1*) interface. For the water splitting reaction path, similar TS were determined for the Pt_3_/TiO_2_(*0 0 1*) interface, Figure 6a, and Pt_3_/TiO_2_(*1 0 1*) interface, Figure 6b. It is possible to observe that the water molecule is dissociated on the Pt_3_/TiO_2_(*0 0 1*) interface, with a 2.0543 Å OH^*^ – Pt bond length and a 1.6364 Å H–Pt bond length. In contrast, for the Pt_3_/TiO_2_(*1 0 1*) interface, the water molecule is dissociated with a slightly longer *OH* ^*^–Pt bond length of 2.0792 Å and a slightly shorter H–Pt bond length of 1.6244 Å. These bond lengths suggest a subtle similar in interaction strength between *OH* ^*^ and Pt across the two interfaces. The Δ*E* calculation indicates that the water molecule splitting exhibits an endothermic character, with Δ*E* = 0.60*eV* for the Pt_3_/TiO_2_(*0 0 1*) interface and Δ*E* = 0.69*eV* for the Pt_3_/TiO_2_(*1 0 1*) interface, suggesting a slightly favorable dissociation process. However, the determined *E_a_* values for the Pt_3_/TiO_2_(*0 0 1*) interface and Pt_3_/TiO_2_(*1 0 1*) interface are nearly identical at 1.39 and 1.40 eV, respectively.

The literature does not report water splitting on the Pt_3_/TiO_2_(*h k l*) interfaces. However, Kong et al. reported that other types of Pt/TiO2 interfaces are discussed,^71^ who studied the Pt/TiO_2_/Ni­(OH)_2_ structure applied in water electrolysis. The authors^71^ performed a theoretical study with DFT and reported a Pt/TiO_2_(*1 0 1*) interface, where the activation energy for water splitting was 0.22 eV. The chosen reaction mechanism involved adsorbing the water molecule on the TiO_2_(*1 0 1*) surface, with the *OH* ^*^ specie adsorbed on the TiO_2_(*1 0 1*) surface, and the H^*^ species adsorbed on the Pt surface. On the other hand, Dai et al.^72^ studied the Pt/TiO_2_–P25 interface for hydrogen evolution from water splitting. The authors^72^ used Pt_9_ and Pt_13_ clusters for the Pt/TiO_2_–P25 interface, simulating water splitting by adsorbing the water molecule on the Pt phase at the interface limit with the TiO_2_ phase’s hydroxyl-rich surface. According to the authors,^72^ the activation energy was in the range of approximately 0.58 to 1.1 eV. Therefore, when the water splitting reaction occurs on the Pt surface, the trend is for the activation of energy to be higher.

The similar behavior between *E_a_* and Δ*E* of the Pt_3_/TiO_2_(*h k l*) interfaces can be attributed to their electronic properties and the reaction mechanism, mainly at the transition state (TS). In this state the H^*^ species is adsorbed on the same Pt atom as the OH^*^ species, after which the H^*^ undergoes diffusion via the spillover mechanism. However, the PDOS analysis provides further into the similar behavior observed for the Pt_3_/TiO_2_(*h k l*) interfaces, as shown in Figure 7.

**Figure 7:**
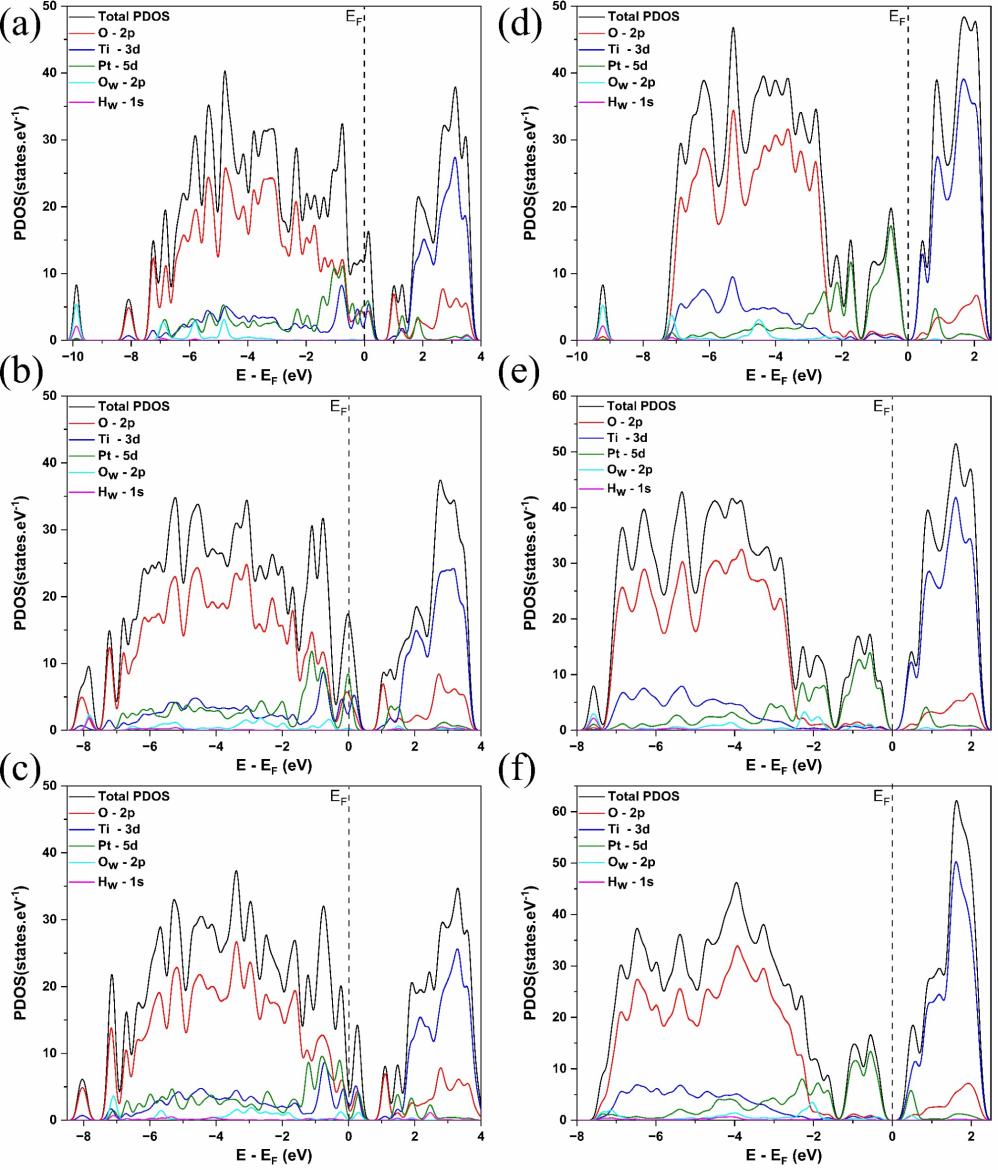
Projected density of states (PDOS) for water splitting on the Pt_3_/TiO_2_(0 0 1) interface (a) IS, (b) TS, (c) FS, and for Pt_3_/TiO_2_(1 0 1) interface (d) IS, (e) TS, and (f) FS.

Figure 7a–f shows that the Pt–5*d* states, green lines in PDOS, are localized near the Fermi energy (*E*_*F*_). In the IS of the Pt_3_/TiO_2_(*0 0 1*) interface (Figure 7a), the O_w_–2*p* and H_w_–1*s* states of the water molecule (cyan and purple lines in PDOS, respectively) are localized around −10 eV and within the range of −7 eV to −4 eV. Similarly, in the IS of the Pt_3_/TiO_2_(*1 0 1*) interface (Figure 7d), the O_w_–2*p* and H_w_–1*s* states of the water molecule appear at approximately −9 eV and span the range of −7 eV to −4 eV. The similarity in these energy levels suggests comparable initial state interactions between the water molecule and both interfaces.

In the TS of the Pt_3_/TiO_2_(*0 0 1*) interface, as shown in Figure 7b, the water molecule undergoes dissociation, with its O_w_–2p and H_w_–1s states shifting to higher energy levels compared to the IS. These states become around −8 eV and within the range of −3 eV to −1 eV, overlapping with the Pt–5*d* state. A similar trend is observed for the TS of the Pt_3_/TiO_2_(*1 0 1*) interface (Figure 7e), where O_w_–2*p* and H_w_–1*s* states are positioned above −8 eV and span the range of −4 eV to −2 eV, also overlapping with the Pt–5*d* state. This overlap suggests interactions between Pt and the dissociated species, which may influence the activation energy. The similarity in electronic structure between the two interfaces indicates that a comparable amount of energy is required for water splitting to proceed, leading to nearly similar *E*_*a*_. However, adsorption geometry may also contribute to this similarity, reinforcing the comparable reaction barriers observed for both surfaces.

In the FS of the Pt_3_/TiO_2_(*0 0 1*) interface, shown in Figure 7c, the O_w_–2*p* and H_w_–1*s* from the water molecule are localized at a lower energy level compared to the TS, around −7 eV, with H_w_–1s states spanning the range of −4 eV to −2 eV. A similar electronic structure is observed in the FS of the Pt_3_/TiO_2_(*1 0 1*) interface, shown in Figure 7f, where the O_w_–2*p* and H_w_–1*s* remain localized around −7 eV, also ranging from −4 eV to −2 eV. The PDOS structure comparison among the IS, TS, and FS for the Pt_3_/TiO_2_(0 0 1) and Pt_3_/TiO_2_(1 0 1) interfaces indicates that the water splitting reaction follows a similar energetic pathway, consistent with the previously determined activation energy. However, in both cases, the reaction on the Pt_3_ cluster surface remains unfavorable due to its endothermic nature and the high activation energy required, which is influenced by both electronic structure and adsorption geometry.

The PDOS structure of water splitting on the Pt_3_/TiO_2_(*h k l*) interfaces provides insights into how the electronic states of water and the interface follow along the reaction. Additionally, the behavior of the *d*-band plays a fundamental role in understanding the interaction between the Pt_3_ cluster and adsorbates, influencing the stability of intermediates during water splitting. Table 2 shows the *d*-band center (ε_*d*_), *d*-bandwidth (*w*_*d*_), and Fermi energy (*E*_*F*_) levels of the isolated Pt_3_/TiO_2_(*h k l*) interfaces, initial state (IS), transition state (TS), and final state (FS) of water splitting on the Pt_3_/TiO_2_(*h k l*) interfaces.

**Table 2:** Electronic Structure of the D-Band Projected: D-Band Center (ε_d_), D-Band Width (w_d_), and Fermi Energy (E_F_) of the Pt_3_/TiO_2_(h K L) Interface Isolate Phase and Water Splitting States on the Pt_3_/TiO_2_(h K L) Interface

	Pt_3_/TiO_2_(*0 0 1*)		Pt_3_/TiO_2_(*1 0 1*)	
*d*-band shape	Ti *d*-band	Pt *d*-band	Ti *d*-band	Pt *d*-band
*ε*_*d*_	–3.37	–2.93	–4.84	–2.05
*w*_*d*_	4.88	2.56	5.44	1.78
*E*_*F*_	–1.74		–0.44	
*Initial state*				
*ε*_*d*_	–4.54	–3.1	–6.49	–2.12
*w*_*d*_	6.22	3.12	6.93	2.04
*E*_*F*_	–1.4		–0.12	
*Transition state*				
*ε*_*d*_	–4.53	–3.32	–6.57	–2.40
*w*_*d*_	6.20	3.18	6.97	2.28
*E*_*F*_	–1.65		–0.3	
*Final state*				
*ε*_*d*_	–4.40	–3.17	–6.53	–2.49
*w*_*d*_	6.22	3.17	7.01	2.23
*E*_*F*_	–1.87		–0.47	

It is possible to notice in Table 2 that in the IS, all ε_*d*_ values shift to lower energy levels relative to the isolated Pt_3_/TiO_2_(*h k l*) interfaces. Additionally, an increase in *w*_*d*_ values in the IS suggests stronger hybridization between Pt and the adsorbed water molecular. The variation in ε_*d*‑Pt_ between the IS and FS is minimal (0.17 eV for Pt_3_/TiO_2_(*0 0 1*) and 0.07 eV for Pt_3_/TiO_2_(*1 0 1*)), indicating that water adsorption does not significantly alter the electronic structure of Pt_3_. In the TS, the *w*_*d*‑Pt_ value for Pt_3_/TiO_2_(*0 0 1*) interface is 3.18 eV, while for Pt_3_/TiO_2_(*1 0 1*) interface, it is 2.28 eV. The narrower *d-*bandwidth in the latter suggests better catalytic selectivity during the water splitting reaction. However, despite the different ε_*d*‑Pt_ variation in this TS (0.22 eV for Pt_3_/TiO_2_(*0 0 1*) and 0.28 eV for Pt_3_/TiO_2_(*1 0 1*)), the *E_a_* remain similar, indicating that water splitting is equally energetically unfavorable for both interfaces. In the FS, the ε_*d*‑Pt_ value of −3.17 eV for the Pt_3_/TiO_2_(*0 0 1*) interface suggests greater stability compared to −2.49 eV for the Pt_3_/TiO_2_(*1 0 1*) interface, though this is noted as a general stability observation rather than a direct electronic effect. In contrast, the variation of the ε_*d*‑Pt_ FS compared with ε_*d*‑Pt_ TS, is 0.15 eV for Pt_3_/TiO_2_(*0 0 1*) interface and 0.09 eV for Pt_3_/TiO_2_(*1 0 1*) interface. Therefore, as shown in the reaction path (Figure 6), the PDOS structure (Figure 7a–f), and the *d*-band analysis (Table 2), water splitting on the Pt_3_ surface of the Pt_3_/TiO_2_(*h k l*) interfaces will not proceed the spontaneous process.

The Pt/TiO2 structure is widely used as a photocatalyst for hydrogen production from water splitting and has been reported to achieve high hydrogen generation rates (HGR) in the literature, ranging from ∼325 to 1023 (μmol.h^–1^).^73–76^ However, other catalyst structures have demonstrated even higher HGR values, such as Fe_3_O_4_@C@TiO_2_ (1593 μmol.h^–1^),^77^ Cu/TiO_2_ (2061 μmol.h^–1^),^78^ and Cu/Ni@Ni/TiO_2_ (13,450 μmol.h^–1^).^79^ This comparison highlights a limitation in the HGR efficiency of the Pt/TiO_2_ interface, such as Pt_3_/TiO_2_(*h k l*) interfaces. The water splitting reaction can occur either on the Pt surface or TiO_2_(*h k l*) surface; however, as shown in this study, water splitting on the Pt is not favorable, as shown here, water splitting is not favorable due to both high activation energy (*E*_a_) and the endothermic nature (ΔE) of the reaction. The reaction path analysis reveals similar reaction states for the Pt_3_/TiO_2_(*h k l*) interfaces, indicating a fundamental limitation of the Pt_3_/TiO_2_(*h k l*) interface regardless of surface orientation. The initial state (IS), water adsorbs onto the Pt_3_/TiO_2_(*h k l*) interface, with hydrogen atoms oriented toward the Pt surface. Specifically, in the Pt_3_/TiO_2_(*1 0 1*) interface, one hydrogen atom is positioned near the TiO_2_(*1 0 1*) surface. The transition state (TS) analysis shows similar reaction pathways for both interfaces, with calculated ΔE values confirming the endothermic nature of water splitting. Additionally, the activation energy (*E*_*a*_) remains comparable across the Pt_3_/TiO_2_(*h k l*) interfaces. PDOS analysis provided insights into the interactions between *OH* ^*^ and *H* ^*^ species derived from the water molecule and Pt atoms across IS, TS, and FS, highlighting the energy levels associated with each reaction state on the Pt_3_/TiO_2_(*0 0 1*) interface. Furthermore, *d*-band analysis demonstrated that the Pt_3_/TiO_2_(*1 0 1*) interface has minimal influence on water interaction, as its electronic structure remains largely unchanged upon water adsorption. This reduced interaction has implications for reactivity and activation energy, reinforcing the conclusion that water splitting on the Pt_3_/TiO_2_(*h k l*) interfaces is not a spontaneous process. While the results provide fundamental insights into the adsorption energetics and electronic interactions of isolated H_2_ and H_2_O molecules on the Pt_3_/TiO_2_ surface, this is a simple approach to the complexity of catalytic reactions, used mainly in DFT calculations.^33,80–83^ However, the molecular interactions and coverage effects can change the binding behavior and reaction dynamics.

## 4 Conclusions

The investigation into hydrogen and water splitting on the Pt_3_/TiO_2_(*h k l*) interface is adequate but requires a more comprehensive understanding of the reaction mechanism. The structural and electronic properties of the Pt_3_/TiO_2_(*h k l*) interface and hydrogen and water splitting on the Pt_3_ surface were investigated through DFT/PW to explore the electronic and structural properties of the interface and DFT/CI-NEB to examine reaction pathways and energy barriers.

Structural analysis showed that the Pt_3_/TiO_2_(*h k l*) interfaces exhibit Pt–Pt, Pt–Ti, and Pt–O bonds, with their distribution influencing the interaction strength between Pt_3_ and TiO_2_(*h k l*). In the Pt_3_/TiO_2_(*0 0 1*) interface, a higher number of Pt–O bonds were observed, whereas the Pt_3_/TiO_2_(1 0 1) interface exhibited more Pt–Ti bonds. The band structure (BS) and projected density of states (PDOS) results indicate metallic behavior in both interfaces due to the overlap of valence and conduction bands, leading to electron delocalization. The Pt_3_/TiO_2_(*0 0 1*) interface exhibited a maximum charge density difference (Δ*n*_max_) of ∼1.54, while the Pt_3_/TiO_2_(*1 0 1*) interface presented a Δ*n*_max_ of ∼1.47. The 2D electron localization function (ELF – η­(*r*)) maps showed that the Pt_*3*_/TiO_2_(*0 0 1*) interface exhibits Ti–O bonds with ionic characteristics, while the Pt–O bonds have a more covalent character than Pt–Ti bonds. In contrast, the Pt_3_/TiO_2_(*1 0 1*) interface demonstrated a more covalent character in its Ti–O bonds compared to the Pt_3_/TiO_2_(*0 0 1*) interface, with Pt–O bonds also exhibiting a more covalent character than the Pt–Ti bonds. These bonding differences play a crucial role in influencing the catalytic behavior of the interfaces.

The reaction mechanism for hydrogen molecule splitting begins with the H_2_ molecule in a physiosorbed state at the initial state (IS). In the transition states (TS), the Pt_3_/TiO_2_(*0 0 1*) interface exhibits dissociation of the H_2_ molecule with *E_a_* = 0.36*eV*. In contrast, for the Pt_3_/TiO_2_(*1 0 1*) interface, the H_2_ molecule remains physiosorbed in the TS, with a lower *E_a_* of 0.19*eV*. This suggests that hydrogen splitting is more energetically favorable on the Pt_3_/TiO_2_(*1 0 1*) interface, while the Pt_3_/TiO_2_(*0 0 1*) interface exhibits a more spontaneous process with a smaller reaction energy (Δ*E*). For the water splitting, the reaction mechanism begins with the water molecule absorbing onto the Pt surface of the Pt_3_/TiO_2_(*h k l*) interface in the IS. Both the Pt_3_/TiO_2_(*0 0 1*) and Pt_3_/TiO_2_(*1 0 1*) interfaces exhibit similar TS structures and *E*_*a*_, indicating that water splitting follows the same reaction pathways on both surfaces. The Δ*E* values suggest that the water splitting reaction is endothermic for both interfaces. Therefore, water splitting is not favorable on the Pt_3_/TiO_2_(*h k l*) interface, which may contribute to its limited hydrogen generation rate (HGR) compared to other catalysts reported for water splitting.

## Supplementary Material



## Notes

The characterization X-ray diffraction with Rietveld refinement; structural and statistical parameters obtained from Rietveld refinement; optimized structures and bond lengths; band structure and projected density of states (PDOS); images of the initial, transition, and final states of hydrogen and water splitting; and the atomic coordinates (*x, y, z*) of TiO_2_ and Pt (PDF)
