# Supplementary material for: The Role of Pt3/TiO2(h K l) Interface in Hydrogen and Water Splitting: A DFT Perspective on Reactivity and Limitations
Source: ACS Omega. 2025 Aug 7;10(32):35604–17. doi: 10.1021/acsomega.5c01650 (PMC12368631; doi:10.1021/acsomega.5c01650)
Supplement: Supplementary file 1 [file ao5c01650_si_001.pdf]

The Role of Pt_3/TiO_2(*h k l*) Interface in Hydrogen and Water Splitting: A DFT Perspective on Reactivity and Limitations.

Ericson H. N. S. Thaines[a,b,e*], Aline C. Oliveira[b], Leandro A. Pocrifka[a], Gustavo Doubek[c], Leonardo M. da Silva[d], Hudson Zanin[e], Renato G. Freitas[b*]

[a]Laboratory of Electrochemistry and Energy, Department of Chemistry, Federal University of Amazonas, 69067-005, Manaus - AM, Brazil.

[b]Laboratory of Computational Materials, Institute of Physics & Institut of Chemistry, Federal University of Mato Grosso, 78060-900, Cuiaba - MT, Brazil.

[c]Laboratory of Advanced Batteries, Advanced Energy Storage Division, Center for Innovation on New Energies, School of Chemical Engineering, University of Campinas; 13083-852, Campinas - SP, Brazil.

[d]Department of Chemistry, Laboratory of Fundamental and Applied Electrochemistry, Federal University of Jequitinhonha e Mucuri's Valley, Rodovia MGT 367, km 583, 5000, Alto da Jacuba, 39100-000, Diamantina - MG, Brazil.

[e]Advanced Materials Labs and Manufacturing Group, Advanced Energy Storage Division; Center for Innovation on New Energies; University of Campinas; 13083-852, Campinas - SP, Brazil.

Supplementary Information

*Corresponding authors:

hthaines@unicamp.br

rgfreitas@fisica.ufmt.br

1. X-ray diffraction (XRD) and Rietveld refinement

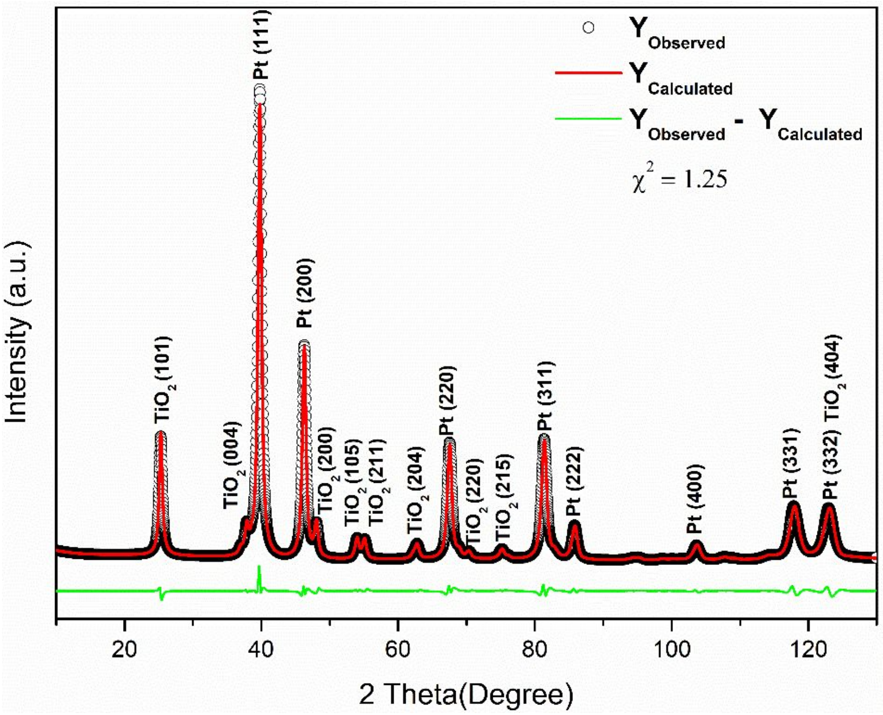

Figure S1. XRD and Rietveld refinement pattern for Pt/TiO_2 crystalline material.

Table S1. Structural and statistical parameters using Rietveld refinement for Pt/TiO_2 crystalline material.

Parameters/phase	Tetragonal/Anatase – TiO_2	Cubic/Pt
a/Å	3.77891(11)	3.91492(3)
b/Å	3.77891 (0)	3.91492 (0)
c/Å	9.4799(5)	3.91492 (0)
$\alpha = \beta = \gamma$ /degrees	90.0	90.0
V/ $Å^3$	135.374(9)	60.0026(15)
Particle size/nm	12.81	12.43
Wt. Fraction	0.59919	0.40081
χ^2	1.25	
$R(F^2)$	0.0211	
wRp	0.0451	
GOF	1.16	

2. Pt_3/TiO_2(*h k l*) interfaces bonds

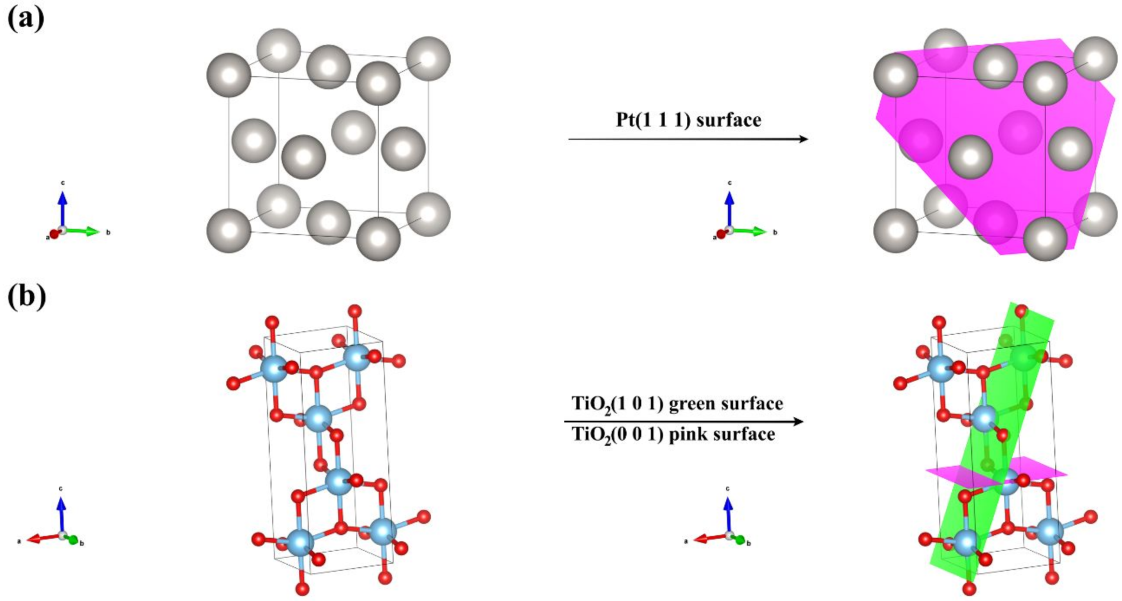

Figure S2 – Structure obtained from CIF file from Rietveld refinement. (a) Gray balls represented the platinum atoms, and (b) red balls represented oxygen atoms and blue balls represented titanium atoms.

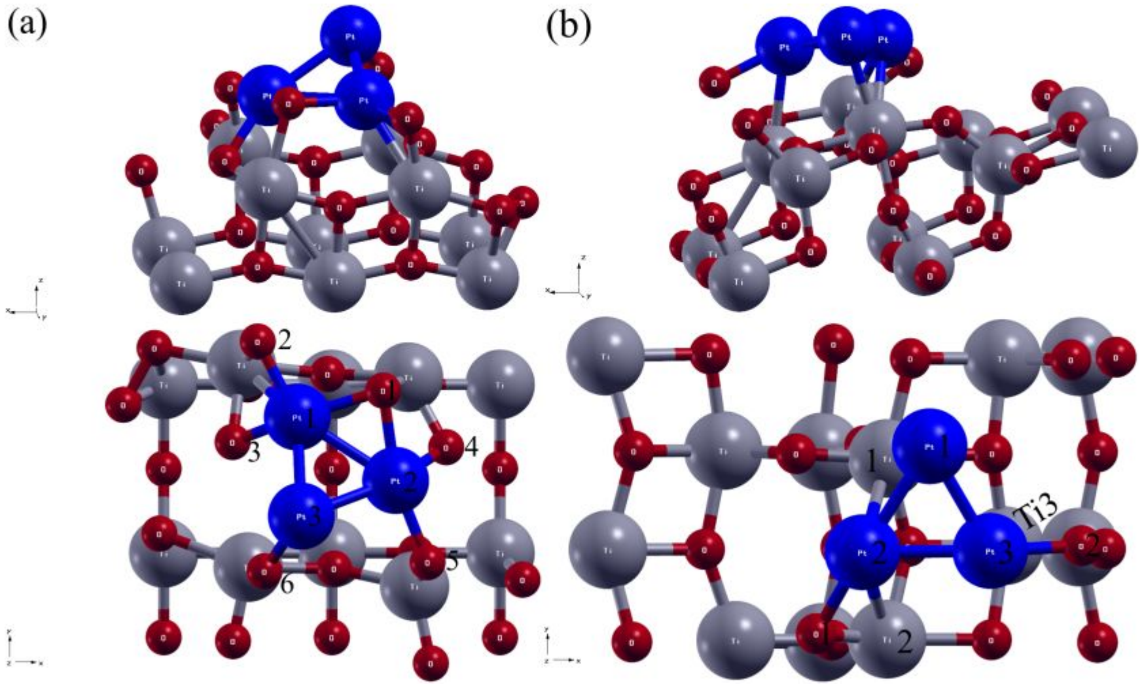

Figure S3. The optimized structures of the (a) Pt_3(*1 1 1*)/TiO_2(*0 0 1*) interface side and top view and (b) Pt_3(*1 1 1*)/TiO_2(*1 0 1*) interface side and top view, respectively.

Table S2 – Bond lengths of the optimized structures of the Pt_3(*1 1 1*)/TiO_2(*h k l*)

**Distance (Å)**	**Pt_3/TiO_2(*0 0 1*)**	**Distance (Å)**	**Pt_3/TiO_2(*1 0 1*)**
Pt1 – Pt2	2.4817	Pt1 – Pt2	2.5438
Pt1 – Pt3	2.6397	Pt1 – Pt3	2.4860
Pt2 – Pt3	2.6042	Pt2 – Pt3	2.5546
Pt1 – $Ti1_{(5C)}$	2.6203	Pt1 – $Ti1_{(5C)}$	2.6372
Pt1 – O1	2.0238	Pt2 – $Ti1_{(5C)}$	2.7836
Pt1 – O2	1.9182	Pt2 – $Ti2_{(4C)}$	2.7145
Pt1 – O3	2.0035	Pt3 – $Ti3_{(5C)}$	2.8049
Pt2 – O1	2.0568	Pt2 – O1	2.0643
Pt2 – O4	1.9955	Pt3 – O2	2.0206
Pt2 – O5	1.9848	-	-
Pt3 – O6	1.8569	-	-

3. Band Structure and Projected Density of States (PDOS)

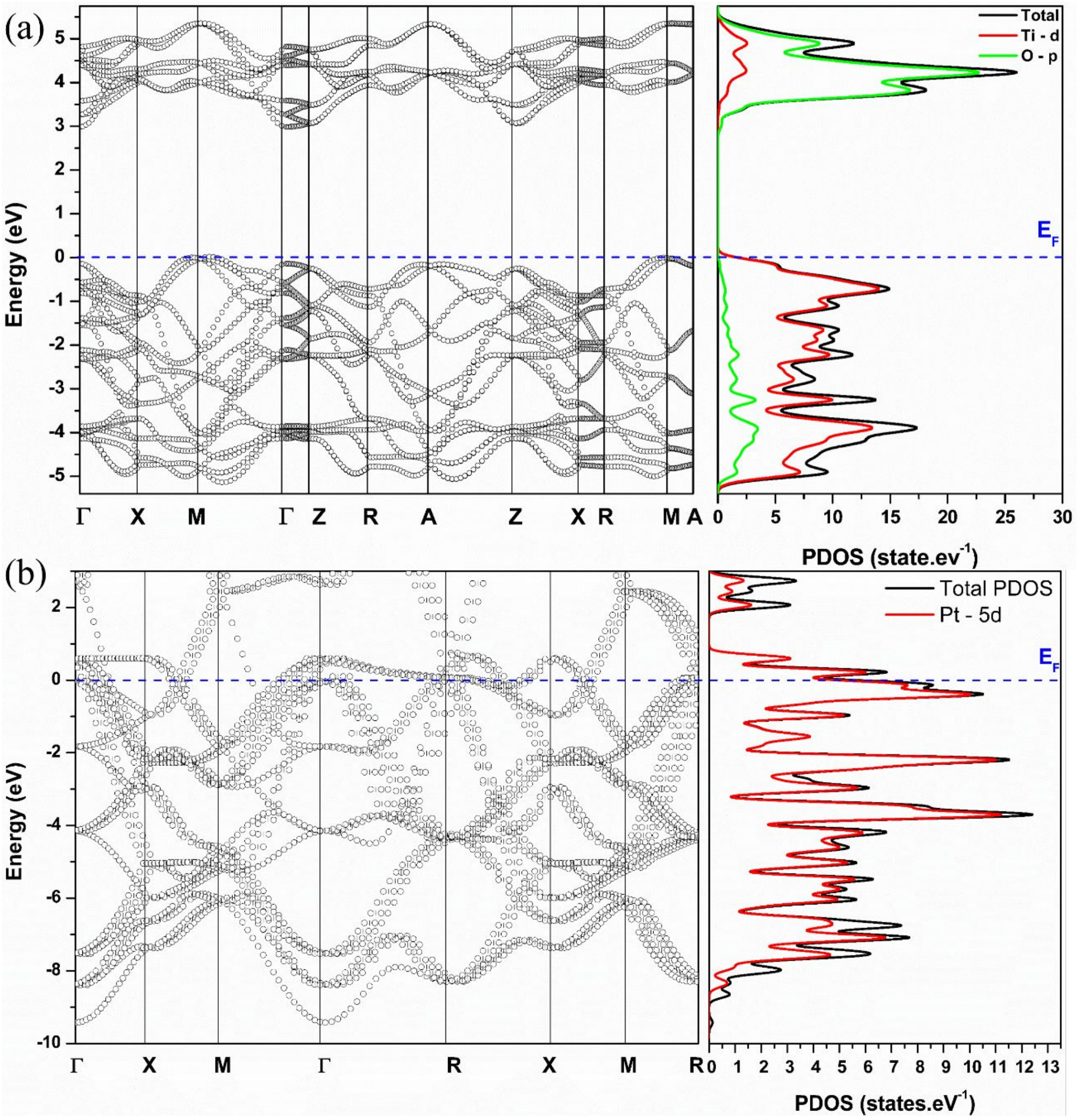

Figure S4 – Band Structure and Projected Density of States (PDOS) for (a) anatase TiO_2 and (b) Pt from Rietveld refinement structure.

4. Images of the initial, transition, and final states of hydrogen and water splitting reactions.

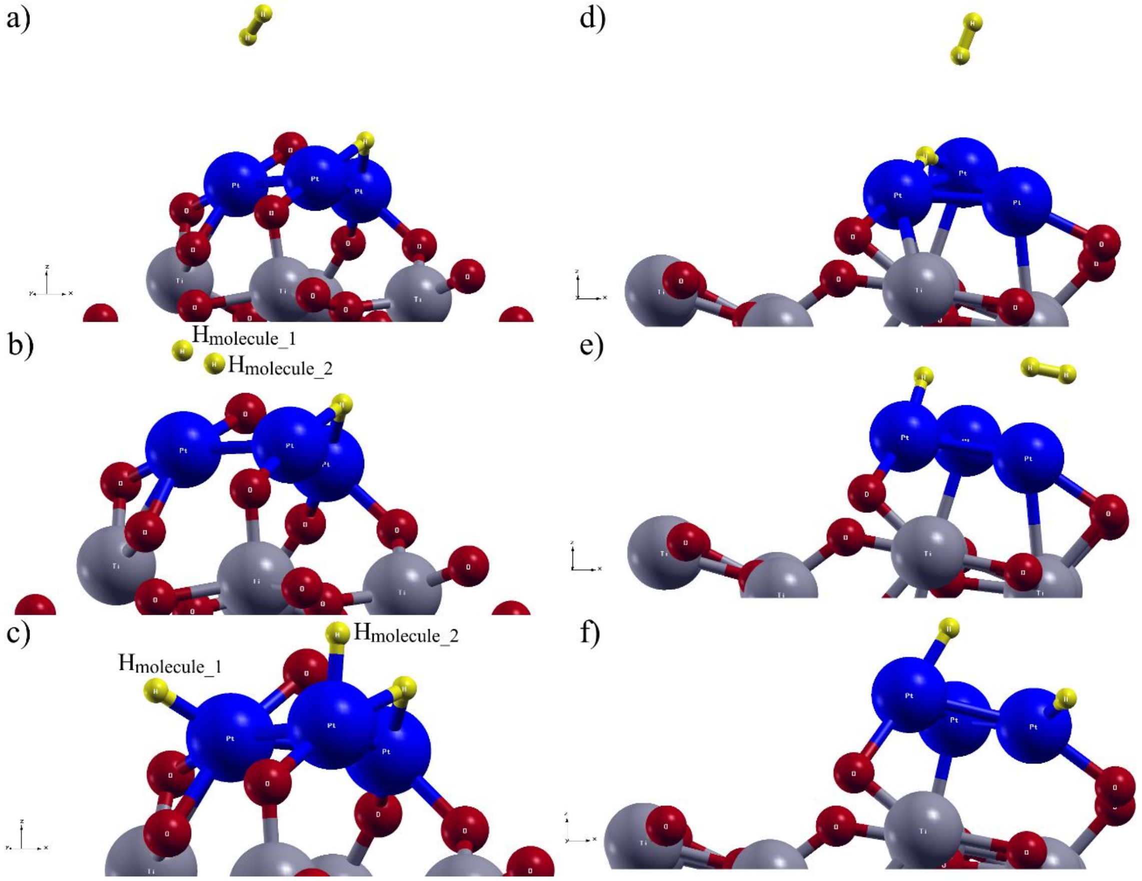

Figure S5 - Images of initial, transition, and final states of hydrogen splitting reactions a), b), and c) Pt_3/TiO_2(*0 0 1*), d), e), and f) Pt_3/TiO_2(*1 0 1*).

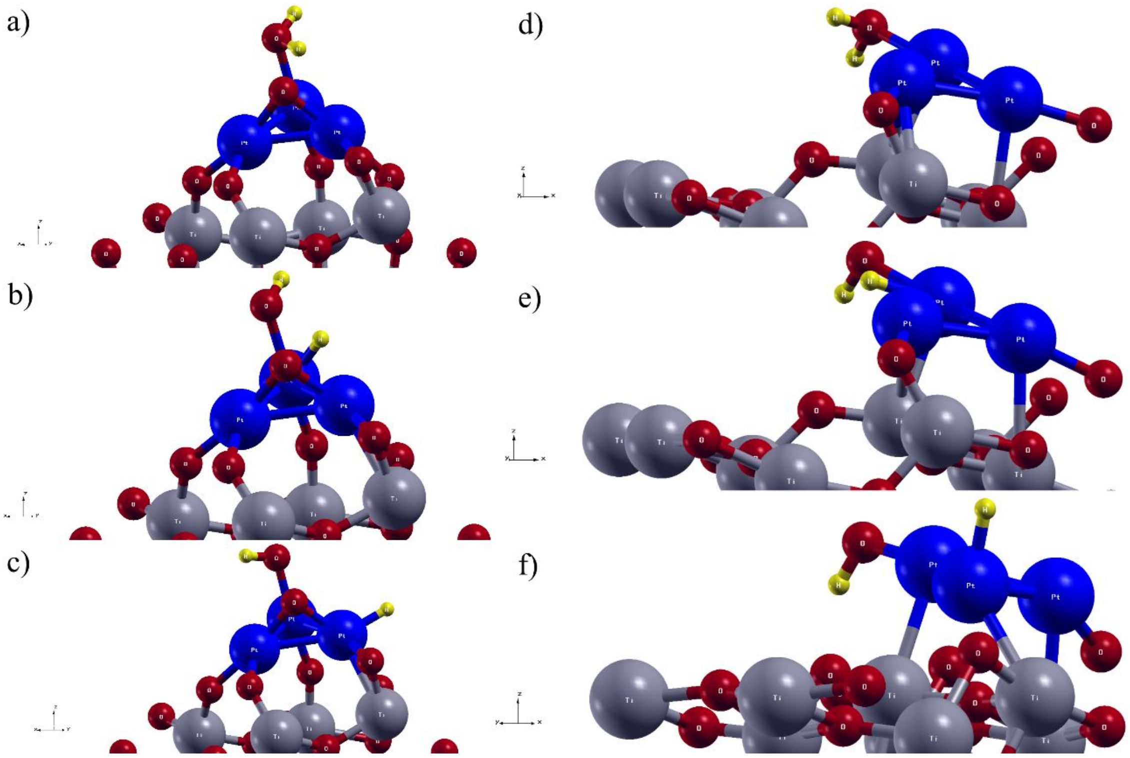

Figure S6 - Images of initial, transition, and final states of hydrogen splitting reactions a), b), and c) Pt_3/TiO_2(0 0 1), d), e), and f) Pt_3/TiO_2(1 0 1).

5. Coordinates (x y z)

Table S3 – Coordinates (*x y z*) of the TiO_2 structure obtained from Rietveld refinement.

Ti	-0.00000	283.966	118.776
O	-0.00000	283.966	317.275
Ti	189.311	0.94655	593.881
Ti	189.311	283.966	356.329
Ti	-0.00000	0.94655	831.434
O	189.311	0.94655	792.380
O	189.311	283.966	554.828
O	-0.00000	0.94655	0.79723
O	189.311	283.966	157.830
O	-0.00000	0.94655	632.935
O	-0.00000	283.966	870.487
O	189.311	0.94655	395.382

Table S4 – Coordinates (*x y z*) of the Pt structure obtained from Rietveld refinement.

Pt	0.00000	0.00000	0.00000
Pt	-0.00000	196.230	196.230
Pt	196.230	-0.00000	196.230
Pt	196.230	196.230	0.00000
